# Collection of human bacteria at University of Tartu, Estonia

**DOI:** 10.1128/mra.00758-25

**Published:** 2025-12-23

**Authors:** Reet Mändar, Hugo Mändar, Tiiu Rööp, Marika Mikelsaar

**Affiliations:** 1 Department of Microbiology, Institute of Biomedicine and Translational Medicine, University of Tartu 37546, Tartu, Estonia; 2 Institute of Physics, University of Tartu 37546, Tartu, Estonia; University of Wisconsin-Madison, Madison, Wisconsin, USA

**Keywords:** lactic acid bacteria, opportunistic bacteria, culturomics, culture collection

## Abstract

The Human Microbiota Biobank (HUMB) at University of Tartu, Estonia, was established to support and promote research activities in the field of human microbiota. The collection contains more than 20,000 bacterial strains, both lactic acid bacteria and opportunistic bacteria. The content of the collection is available for scientific collaboration.

## ANNOUNCEMENT

In 1889, Franz Kral founded the “Kral Bacteriological Museum” in Prague, pioneering microbial culture collections (1). In the University of Tartu (UT), Estonia, studies on human microbial ecology began during the 1960s under Prof. Akivo Lenzner, including research on Soviet cosmonauts’ lactobiota. The cosmonauts received lyophilized strains of their own beneficial lactobacilli, marking the emergence of personalized medicine. During the 1990s, the collected lactobacilli were transferred to a partner institute in Moscow (2). The Human Microbiota Biobank (HUMB) in UT was established by Prof. Marika Mikelsaar in 1994 during the Estonian-Swedish allergy study (3). In addition to lactobacilli, opportunistic bacteria were added to collection through several national and EU projects (4–7). In 2010, the HUMB joined the World Data Center for Microorganisms (WDCM), and in 2012, the European Cultural Collections Organization (ECCO).

**Fig 1:**
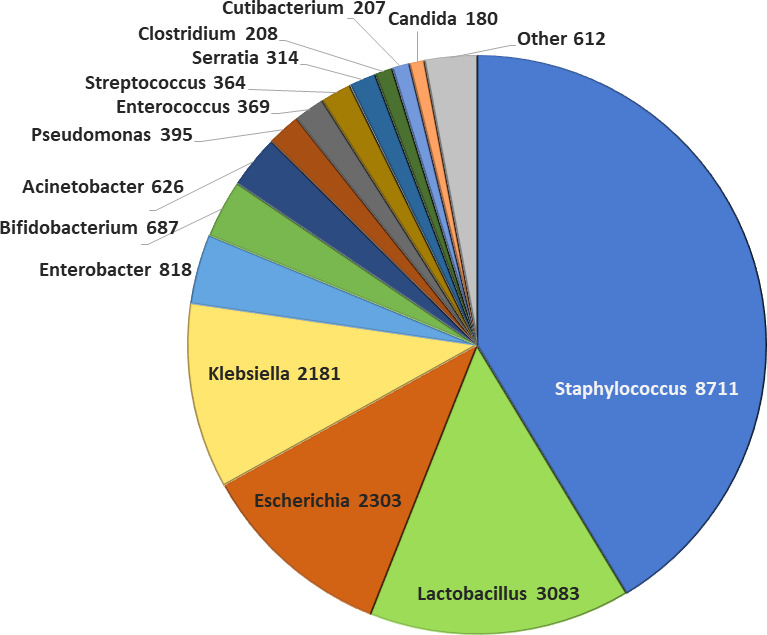
Number of strains of the most numerous genera in the HUMB collection.

As of 2025, the collection holds nearly 20,000 human-derived microbial strains from over 120 genera and 350 species (Fig. 1). The HUMB contains both lactic acid bacteria and opportunistic bacteria, many of which have been characterized for properties and genetic traits.

Lactic acid bacteria in the HUMB form an important basis for probiotic development. Patented strains (deposited also in DSMZ) include *L. fermentum* ME-3 (8–14) developed in University of Tartu and *L. plantarum* Tensia (15) and *L. plantarum* Inducia (16) developed in a competence center, BioCC. ME-3 is an antioxidant and anti-inflammatory strain; Tensia lowers diastolic blood pressure through various mechanisms; and Inducia lowers total and ox-LDL cholesterol levels. These strains are available in several food and pharmaceutical products.

Opportunistic bacteria in the HUMB originate from different specimens of healthy and diseased subjects (Table 1). In the majority of them, properties have been detected like antibiotic susceptibility, resistance-associated genes, or typing patterns (17–19).

**TABLE 1:** Subcollections of bacteria in the HUMB collection

Subcollection	Project	Acronym	Strains	References^*a*^
Lactic acid bacteria	Estonian-Swedish allergy study	Est-Swe allergy	778 strains of lactobacilli were collected in the cohort of Estonian and Swedish children aged 1 week to 5 years in 1995–2004. The following species are more numerous: *Lactobacillus acidophilus* (*n* = 56), *Lactobacillus fermentum* (*n* = 55), *Lactobacillus casei* (*n* = 348), and *Lactobacillus plantarum* (*n* = 81).	(3, 20–26)
	*Clostridium difficile* infection in Estonia and Norway: molecular epidemiology (including prevalence of hypervirulent strain 027), phenotypic characteristics of strains and interaction with intestinal lactobacilli	Estonia-Norway	More than 200 strains of lactobacilli were collected from hospitals in Tallinn, Tartu, and Stavanger in 2009–2010. The following species are more numerous: *Lactobacillus rhamnosus* (*n* = 36), *Lactobacillus plantarum* (*n* = 15), and *Lactobacillus casei* (*n* = 11).	(27, 28)
	Innovative research for milk products of additive value	ME-3 kefir trial	2,230 strains were isolated in 2013–2014. The subcollection contains isolates from more than 100 species, including 27 species of lactobacilli, nine species of bifidobacteria, and 13 species of Clostridium. The following species are more numerous: *Bifidobacterium adolescentis* (*n* = 160), *Escherichia coli* (*n* = 547), *Lactobacillus acidophilus* (*n* = 56), *Lactobacillus gasseri* (*n* = 238), *Lactobacillus paracasei* (*n* = 111), *Lactobacillus rhamnosus* (*n* = 55), and *Lactobacillus ruminis* (*n* = 117).	(29)
	Rapid induction of passive immunity against weapons of bioterrorism using transformed GRAS (generally regarded as safe) microorganisms	Biodefense	724 strains from 17 species of lactobacilli were isolated during the international project Biodefense in 2006–2007. The following species are more numerous: *Lactobacillus gasseri* (*n* = 166), *Lactobacillus acidophilus* (*n* = 124), *Lactobacillus paracasei* (*n* = 208), *Lactobacillus rhamnosus* (*n* = 36), and *Lactobacillus ruminis* (*n* = 35). Six strains were used in the frame of the next EU project Lactobody in order to develop transformed lactobacilli for fighting against antibiotic-associated diarrhea.	(30)
	100-year-old bacteria for the health of Estonians	EV100	Fecal samples were collected from centenarians (*n* = 25; aged 96 to 103 years, median 99 years) and young people (*n* = 25; aged 19 to 23 years, median 21 years). Strains from 94 species were isolated, including 10 species of bifidobacteria, 19 species of lactobacilli, and 11 species of clostridia. The most numerous additions to the collection were isolates from the following species: *Bifidobacterium adolescentis* (*n* = 10), *Bifidobacterium longum* (*n* = 26), *Clostridium perfringens* (*n* = 13), *Escherichia coli* (*n* = 111), *Lacticaseibacillus paracasei* (*n* = 12), *Lacticaseibacillus rhamnosus* (*n* = 17), and *Lactobacillus gasseri* (*n* = 12).	(31)
	Oral bacteria in chronic periodontitis and periodontal health	Periodontitis	During the study, 115 strains of 11 species of lactobacilli were isolated. The most numerous were *Lactobacillus casei* (*n* = 14), *Lactobacillus fermentum* (*n* = 29), *Lactobacillus gasseri* (*n* = 15), *Lactobacillus oris* (*n* = 14), *Lactobacillus plantarum* (*n* = 19), and *Lactobacillus rhamnosus* (*n* = 10).	(32, 33)
Staphylococci and other bacteria related to neonatal intensive care units	Optimization of empiric antibacterial therapy for early onset neonatal sepsis	EOS	2,932 strains from more than 40 species were isolated. The following species are more numerous: *C. albicans* (*n* = 136), *E. cloacae* (*n* = 386), *E. coli* (*n* = 196), *K. oxytoca* (*n* = 314), *K. pneumoniae* (*n* = 364), *S. aureus* (*n* = 96), *S. epidermidis* (*n* = 222), and *S. haemolyticus* (*n* = 170).	(34–37)
	Coagulase-negative staphylococci in the gut of preterm neonates and in the breast milk of their mothers	CoNS	A total of 4,749 strains were isolated. The following gram-positive species are more numerous: *S. aureus* (*n* = 119), *S. capitis* (*n* = 175), *S. epidermidis* (*n* = 2,891), *S. haemolyticus* (*n* = 583), and *S. hominis* (*n* = 242). In addition, gram-negative bacteria of more than 30 species were isolated from the same samples, including *E. cloacae* (*n* = 128), *E. coli* (*n* = 162), *K. oxytoca* (*n* = 127), and *K. pneumoniae* (*n* = 46).	(38, 39)
	The role of individual and environmental factors in the colonization of newborns and their impact on the prevention of invasive infections	Preterm infect	3,822 strains were isolated. Gram-positive bacteria included *S. aureus* (*n* = 241), *S. epidermidis* (*n* = 1814), *S. haemolyticus* (*n* = 841), and *S. hominis* (*n* = 275). Gram-negative bacteria included *E. cloacae* (*n* = 81), *E. coli* (*n* = 119), *K. oxytoca* (*n* = 105), and *K. pneumoniae* (*n* = 36).	(40)
Strains from antibiotic resistance studies	Epidemiology of newer beta-lactamases in the Baltic region and evaluation of possibilities for their real-time molecular screening	BEEP	ESBL-positive *E. coli* (*n* = 477) and *K. pneumoniae* (*n* = 587) were collected from 20 hospitals in Estonia, Latvia, Lithuania, and St. Petersburg region in Russia in 2011–2012.	(41, 42)
	Baltic Antibiotic Resistance Collaborative Network	BARN	1,165 strains belonging to 27 species were collected from nine countries: Finland, Estonia, Latvia, Lithuania, Russia (St. Petersburg), Poland, Belarus, Ukraine, and Georgia in 2012–2015. The following species are more numerous: *E. coli* (*n* = 119), *K. pneumoniae* (*n* = 309), and *P. aeruginosa* (*n* = 248).	(19)
	Routes for development and spread of antibiotic resistance and resistance containment measures	AMR-RITA	404 strains were collected during 2019–2022 include *E. coli* (*n* = 222), *S. aureus* (*N* = 29), and *K. pneumoniae* (*n* = 118).	
Diverse bacteria from various projects	Dental health in preschool and schoolchildren in relation to dental fear and fear-related factors and the outcome of caries prevention program in offspring of fearful mothers	Mutans streptococci	110 isolates from six species were added to the HUMB collection, with the most numerous being *Streptococcus mutans* (*n* = 87) and *Streptococcus sobrinus* (*n* = 19).	(43)
	Etiopathogenesis and diagnostics of peritonsillar abscess	PTA	34 species were isolated, including difficult-to-culture anaerobes, such as *Fusobacterium necrophorum* and *Fusobacterium nucleatum*. The most commonly frozen strains included *Streptococcus pyogenes* (*n* = 70), *Streptococcus parasanguinis* (*n* = 16), and *Streptococcus pneumoniae* (*n* = 9).	(44, 45)
	Gymnasium students’ pocket electronics as a potential source of bacteria-carrying drug resistance genes	Mobile phone	80 strains from 22 species were isolated, with species, such as *Acinetobacter lwoffii* (*n* = 11), *Micrococcus luteus* (*n* = 20), and *Staphylococcus epidermidis* (*n* = 10) being the most numerous.	(46)
	Microbiological purity of money in circulation in Estonia	Currency	223 strains from 46 species were isolated, with the most numerous being *Micrococcus luteus* (*n* = 50), *Staphylococcus capitis* (*n* = 12), *Staphylococcus epidermidis* (*n* = 42), *Staphylococcus hominis* (*n* = 12), and *Staphylococcus warneri* (*n* = 17).	(47)
	Impact of probiotics on skin microbiota	Maikli	220 strains belonging to four species were isolated, including *Cutibacterium acnes* (*n* = 171).	
	Microbiota of endometrium in women with and without endometriosis	Culturomics	This international collaboration project between University of Tartu, University of Granada, and Celvia CC added 216 strains from 40 species to the collection. Most numerous species are *Cutibacterium acnes* (*n* = 19), *Gardnerella vaginalis* (*n* = 17), *Lactobacillus crispatus* (*n* = 28), *Lactobacillus gasseri* (*n* = 9), *Lactobacillus iners* (*n* = 10), and *Staphylococcus epidermidis* (*n* = 18).	

^*a*^ Only a few examples of references are given in the table. More references can be seen on the HUMB website.

Collection supports continuing research. Lactic acid bacteria are tested *in vitro* and *in vivo* to develop novel probiotics and functional foods (48, 49). Drug resistance and virulence markers are monitored in opportunistic bacteria. Over 40 PhD and MSc theses have been defended and over 400 papers published using and/or supplementing the collection.

The development of modern database and website was initiated in 2010. The information platform is based on MySQL. The database was developed using dbForge Studio (Devart). PHPRunner (Xlinesoft) was used to create a web-based user interface that is managed by cPanel platform (cPanel, L.L.C.) on a webserver of UT.

The HUMB webpage is hosted by Estonian Electronic Microbial DataBase (EEMB). The other collections within EEMB (CELMS, CREP) are not associated with the HUMB. Guest users can view basic catalog data, while registered users have access to full structure with editing capabilities.

New strains are obtained into collection according to Material Deposit Agreement (MDA) in accordance with ECCO suggestions (50, 51).

Strains are available for academic research under Material Transfer Agreement (MTA). Eligible collaborators include accredited academic institutions, publicly funded research organizations, and non-profit entities engaged in scientific research. Commercial entities may be considered on a case-by-case basis, subject to separate agreements. All collaborations are expected to result in shared scientific outputs, such as publications, dissertations, or patents.

## Data Availability

The webpage of the HUMB collection is https://eemb.ut.ee/eng/humb_english_introduction_list.php. The catalogue of the HUMB collection may be accessed at https://eemb.ut.ee/humb/login.php (username, guest; password, guest). Accession numbers for the publicly available sequence data may be found at https://datadoi.ee/handle/33/698. The collection is open for scientific collaboration. Strains are available for academic research under a Material Transfer Agreement. The MTA contains a statement that the material must be handled with appropriate care, as not all of its properties are known. In addition, by signing the MTA, the recipient confirms that they possess the necessary expertise, facilities, and legal rights to handle the materials listed therein. Eligible collaborators include accredited academic institutions, publicly funded research organizations, and non-profit entities engaged in scientific research. Commercial entities may be considered on a case-by-case basis, subject to separate agreements. All collaborations are expected to result in shared scientific outputs, such as publications, dissertations, or patents. For requests for the resource and additional information, please contact Tiiu Rööp (tiiu.roop@ut.ee) or Reet Mändar (reet.mandar@ut.ee).
